# Lower Extremity Muscle Activation in Alternative Footwear during Stance Phase of Slip Events

**DOI:** 10.3390/ijerph18041533

**Published:** 2021-02-05

**Authors:** Harish Chander, John C. Garner, Chip Wade, Adam C. Knight

**Affiliations:** 1 Neuromechanics Laboratory, Department of Kinesiology, Mississippi State University, Mississippi State, MS 39762, USA; aknight@colled.msstate.edu; 2 Department of Health and Kinesiology, Troy University, Troy, AL 36082, USA; jcgarner@troy.edu; 3 Department of Industrial and Systems Engineering, Auburn University, Auburn, AL 36849, USA; lrw0002@auburn.edu

**Keywords:** unexpected slips, alert slips, expected slips, flip-flops, Crocs, slip-resistant shoes

## Abstract

Muscle activity from the slipping leg have been previously used to analyze slip induced falls. However, the impact of casual alternative footwear on slipping leg muscle activity when exposed to slippery environments is still unknown. The purpose of the study was to analyze the impact of alternative footwear (crocs (CC) and flip-flops (FF)) compared to slip-resistant footwear (LT) on lower extremity muscle activity when exposed to dry gait (NG), unexpected (US), alert (AS), and expected slips (ES). Eighteen healthy males (age: 22.3 ± 2.2 years; height: 177.7 ± 6.9 cm; weight: 79.3 ± 7.6 kg) completed the study in a repeated measures design in three footwear sessions separated by 48 h. Electromyography (EMG) muscle activity from four muscles of the lead/slipping leg was measured during the stance phase of the gait-slip trials. A 3 (footwear) × 4 (gait-slip trials) repeated measures analysis of variance was used to analyze EMG dependent variables mean, peak, and percent of maximal voluntary contraction. Greater lower extremity muscle activation during the stance phase was seen in US and AS conditions compared to NG and ES. In addition, footwear differences were seen for the alternative footwear (CC and FF) during US and AS, while the low top slip resistant shoe had no differences across all gait trials, suggesting it as the most efficient footwear of choice, especially when maneuvering slippery flooring conditions, either with or without the knowledge of an impending slip.

## 1. Introduction

The failure of normal locomotion and attempts at equilibrium recovery following induced imbalance leads to slips, trips, and falls [1,2]. Postural instability can lead to an increased risk of falls, slips, trips, and other accidents [3]. Electromyography (EMG) analysis has been used to analyze neuromuscular mechanisms in human balance and gait. Normal human locomotion consists largely of eccentric and/or isometric muscle action of the lower extremity for efficient storage and transfer of energy between limb segments with periods of concentric muscle actions that help in the forward motion of the body. However, lower extremity muscle activity during slips and slip recovery are different from muscle activation patterns during normal dry locomotion.

Muscle activity that is accountable for the reactive and proactive lower extremity moments are crucial factors to control human posture during slips and impending slips [4]. Muscle activity for quadriceps, hamstrings, and gastrocnemius-soleus have been commonly reported under slippery conditions [5,6,7]. Longer muscle activation periods with higher magnitudes have been reported during slips [8,9,10]. A longer hamstring activity and a lower quadriceps activity during the stance phase has been reported during slippery gait [6]. Furthermore, lower mean and peak swing leg gastrocnemius activity was also reported during slippery conditions [6]. Similar muscular responses were also seen under slip events when compared with young and old aged individuals, with a delayed latency from vastus lateralis activity in severe slips [5]. Corrective muscular responses have been shown to produce large moments at the knee joint when recovering from a slip, while the hip joint moments play a larger role in stabilization [7]. Muscle activity in the hamstring had the greatest increase out of the lower extremity musculature, while the gastrocnemius had the earliest muscle activation during an alert or expected [5]. Activation of agonist/antagonist pairs of tibialis anterior and medial gastrocnemius and vastus lateralis and medial hamstrings were found to be greater when awaiting a slippery surface and individuals with a larger co-contraction while walking were prone to less severe slips [5]. Furthermore, different types of footwear have been shown to impact normal balance and gait mechanisms [11,12,13,14,15,16,17,18,19]. Commonly worn footwear such as slippers have been found to be hazardous as they slowed down reactions to perturbations and also had adverse effects on posture reactions [20,21]. Although there is an increasing amount of literature assessing the impact of different types of footwear including alternative footwear on human locomotion, there is still a dearth of literature on the impact of these types of footwear on slippery conditions [21], especially when exposed to different types of slips such as without and with the knowledge of an impending slip.

More recently, the current researchers have investigated the impact of alternative footwear such as Crocs and flip-flops during various slip events. Alternative footwear has been reported to increase the severity of slips experienced when the slip was unexpected based on kinematic [22] and kinetic analysis [23]. The alternative footwear (Crocs and flip-flops) exhibited greater incidence of hazardous and potentially hazardous slips, when compared to slip-resistant footwear, especially during unexpected slips. Additionally, from the same study, when kinematics, kinetics, and muscle activity were analyzed during slip initiation (120 ms post-heel strike), increased plantar flexion angles, lower vertical ground reaction forces, and greater lower extremity muscle activity were identified in these alternative footwear (Crocs and flip-flops) during unexpected, alert, and expected slip trials when compared to slip-resistant footwear and dry normal gait [4]. It is also suggested that wearing alternative footwear such as flip flops, sandals, and Crocs decreases an individual’s movement ability and increases the required muscle activity from the lower extremity due to the hind foot not being secure and does not move with the foot as one rigid segment [4,24]. While the slip severity [22], ground reaction forces during stance phase [23] as well as joint kinematics, ground reaction forces, and muscle activity during slip initiation [4] from the current study have been previously reported and published, lower extremity muscle activity during the entire stance phase of the gait cycle has not been analyzed yet. Hence, to gain an understanding of the corrective and reactive muscle activity responses during slippery gait in alternative footwear, analysis of previously unreported data from the same study on lower extremity muscle activity during the entire stance phase of the gait and slip trials from the same study is warranted. Therefore, the specific purpose of this paper was to analyze the impact of alternative footwear (Crocs with clogs (CC), flip-flops (FF) and low top slip resistant shoe (LT)] under multiple gait conditions (dry normal surface (NG); unexpected slip (US), alert slip (AS), and expected slip (ES)) on lower extremity muscle activity. Based on the previous literature, that report slowed responses in alternative footwear and based on the previously reported data from the current study suggesting the increased slip hazard with alternative footwear use, it was hypothesized that the alternative footwear (CC and FF) would demonstrate greater muscle activity compared to LT during both normal dry gait conditions and slippery gait conditions. We also hypothesized that the slippery conditions (US, AS, and ES) will demonstrate greater muscle activity compared to the normal dry gait condition (NG).

## 2. Materials and Methods

### 2.1. Participants

Eighteen healthy male participants (age: 22.3 ± 2.2 years; height: 177.7 ± 6.9 cm; weight: 79.3 ± 7.6 kg) completed the study. Participants who had any history of musculoskeletal injuries, cardio-vascular abnormalities, neurological disorders, vestibular disorders, under medication, or any inability to walk and stand without support were excluded from the study. Healthy young adults were specifically chosen for this study, so that any differences observed could be attributed due to the footwear and gait/slip trial types. Participant sample size was based on similar previous studies conducted in our laboratory and based on prior published literature focusing on footwear and slips [12,25,26]. All participants were recruited through flyers approved by the University of Mississippi’s Institutional Review Board (IRB) (IRB Protocol #14-014). All participants read and signed the informed consent and completed a physical activity readiness questionnaire (PAR-Q) to rule out any of the above-mentioned health complications and cleared for participation in the study.

### 2.2. Instrumentation

**Figure 1:**
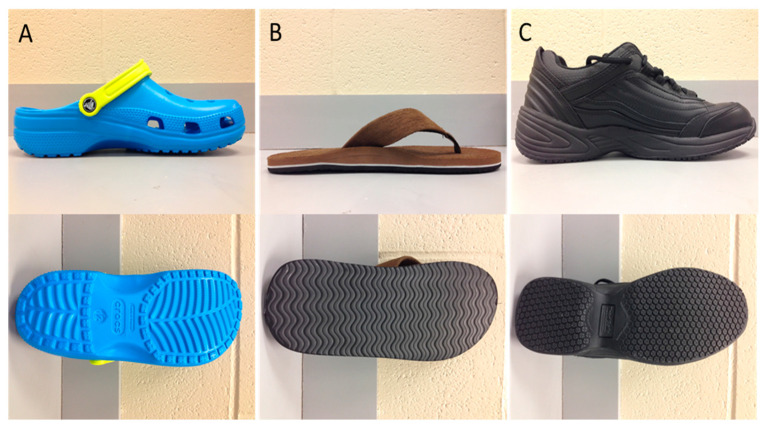
Alternative footwear. (**A**) Crocs with clogs (CC); (**B**) flip-flops (FF) and (**C**) low top slip resistant shoe (LT) exhibiting their overall design and sloe-tread design.

The alternative footwear tested included the CC, FF, and LT (Figure 1). Electromyography (EMG) data were collected using the Noraxon Telemyo DTS 900 system (Scottsdale, AZ, USA) at 1000 Hz. Vicon (Oxford, UK) 3D motion capture system with 12 infra-red T-series cameras was used to collect kinematic gait data using the lower body plug-in gait model from the Helen-Hayes marker system at 100 Hz along with kinetics measured using dual force plates (AMTI and Bertec) at 1000 Hz and synced with EMG. A uni-track fall arrest system from Rigid Lines (Millington, TN, USA) attached to a trolley and a back-pack type harness was used to prevent any falls during slip testing. The slippery agent was composed of 75% industrial vegetable-based glycerol mixed with 25% water based on the previous literature. During the slip gait trials, glycerol was evenly applied on the second force plate, on which the left leg of all participants made contact during the gait and slip trials. To minimize the error due to inter- and intra-rater reliability, the primary investigator always applied the slippery agent using the same measured and calibrated container.

### 2.3. Experimental Procedures

**Figure 2:**
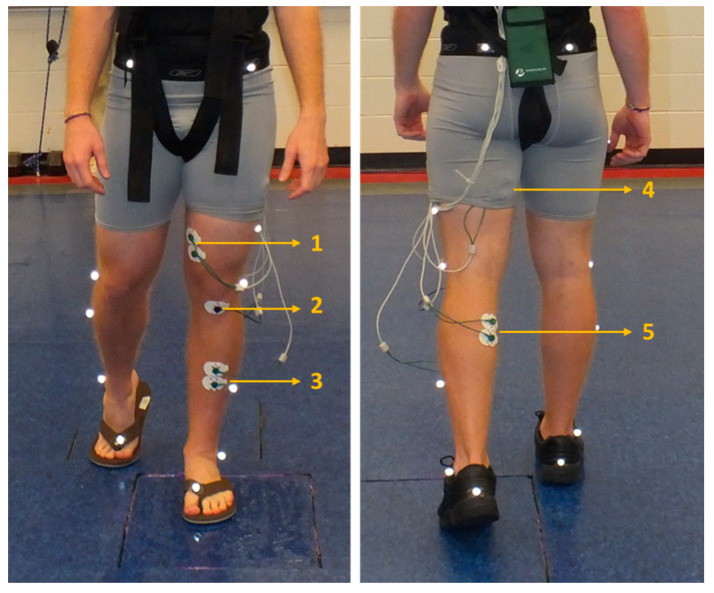
Example of a participant performing the gait-slip trials in flip-flops (FF) (**left**) and low top slip resistant show (LT) (**right**) along with the lower extremity muscles tested. (**1**) Vastus medialis (VM). (**2**) Ground electrode on tibial tuberosity. (**3**) Tibialis anterior (TA). (**4**) Medial hamstrings (MH). (**5**) Medial gastrocnemius (MG).

The study followed a within-subjects repeated measures design, in which each participant was tested on all three footwear types assigned in a counter-balanced order to remove the order effects. After an initial familiarization day, which included anthropometric measurements and three practice trials for measures for gait trials in a safety harness, all participants completed three experimental testing days separated by a minimum of 48 h to prevent any undue muscular fatigue arising out of testing procedures. Participants were also asked to refrain from strength training their lower extremity 24 h before each testing session for the same reason. All three experimental testing days followed the same testing procedures, which started with donning the assigned footwear and placement of EMG bipolar electrodes on the muscle belly of the vastus medialis (VM), medial hamstrings (MH), tibialis anterior (TA), and medial gastrocnemius (MG) on the left leg with an inter-electrode distance of 2 cm while the ground electrode was placed on the tibial tuberosity. These four muscles were chosen to provide a representation of both the upper and lower muscles in the leading/slipping leg [4,27]. The left leg was used as the testing leg or leading leg for all participants as it was the one set-up to strike the longer force platform over which the slippery agent was applied (Figure 2). All participants were left leg non-dominant and testing the left leg provided a consistent approach to slip responses. Prior to placement of the electrodes, the surface area was prepped by shaving hairy surfaces and scrubbing with alcohol rubs to minimize skin resistance. Reflective markers were placed on each participant’s lower extremity and on the footwear following a lower body plug-in gait model from the Helen-Hayes system.

**Figure 3:**
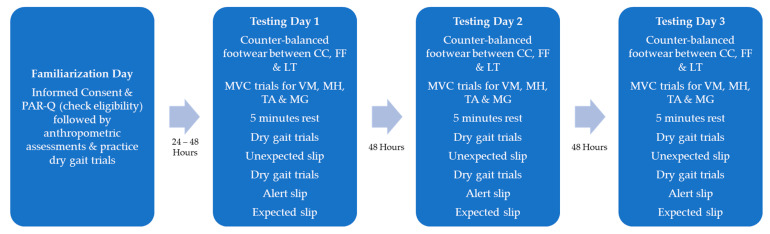
Testing protocol for three footwear types: Crocs (CC), flip-flops (FF), and low-top slip resistant shoe (LT) for four lower extremity muscles: vastus medialis (VM), medial hamstrings (MH), tibialis anterior (TA), and medial gastrocnemius (MG).

The experimental procedures remained the same for all three testing days (Figure 3). Three trials of five second maximal voluntary isometric contractions (MVC) were performed for the VM, MH, TA, and MG in the middle range of motion for the ankle and knee joints. To avoid any undue lower extremity muscular fatigue due to muscular exertion, participants were instructed to rest for 5 min after MVC. Participants were then strapped to the harness and were allowed to practice walking at their self-selected pace across the lab walkway. Participants were instructed to walk as normally as possible with the same speed and a normal dry gait trial was captured. On completion of the normal dry gait trial, participants were faced away from the walking area while listening to music on noise-cancelling headphones for 30–45 s in between further dry gait trials. One particular trial was chosen randomly to be the unexpected slip (US) trial and the glycerol was applied to the force plate without the participant’s knowledge. Participants were still given the same walking instruction to ensure that the walking trial was treated as an unexpected slip event. On completion of the US, participants were allowed to rest briefly, and the footwear and the force plate was and made ready for the next gait trials. Following the US trial, participants performed multiple normal dry gait trials, and once a normal gait pattern resumed, participants were given the instruction that the following trials may or may not be slippery. One trial was randomly chosen to be the alert slip (AS) trial, where the glycerol was applied again without the knowledge of the participant. Finally, with the completion of NG, US, and AS and with the footwear and force plate cleaned, participants were instructed that the following trial would be slippery and performed one slip trial, which was treated as the expected slip (ES). EMG data were collected during all gait trials.

### 2.4. Data Analysis

The EMG raw data were filtered using a Butterworth fourth order filter with zero lag with cut off frequency of 300 Hz and rectified using full wave rectification. The moment of heel strike and toe off for the left leg during the gait trials to determine the beginning and ending of the stance phase was collected using Vicon Nexus software. Mean muscle activity during MVCs (mV) from the four muscles (VM MVC, MH MVC, TA MVC, and MG MVC), mean muscle activity (mV) (mean VM, mean MH, mean TA, and mean MG), peak muscle activity (mV) (peak VM, peak MH, peak TA, and peak MG), and %MVC (%MVC VM, %MVC MH, % MVC TA, and %MVC MG) during the stance phase of all gait trials (NG, US, AS, and ES) were calculated.

### 2.5. Statistical Analysis

A Within-Subjects 3 × 4 [3 footwear (CC, FF, LT) × 4 gait trials (NG, US, AS, ES)] repeated measures analysis of variance (ANOVA) was used to analyze the dependent EMG variables of mean muscle activity, peak muscle activity, and %MVC individually for all four muscles. A Greenhouse Geisser correction was used if the Mauchly’s test of sphericity was violated. If a significant footwear × gait trial interaction existed, the main effects for both footwear and gait trials were discarded and simple effects were analyzed using the Bonferroni Sidak correction. If no interaction was found, the main effect significance was analyzed using pairwise comparisons using the Bonferroni Sidak correction. For all analyses, alpha level was set at *p* < 0.05 and all statistical analyses were performed using SPSS 24.

## 3. Results

**Figure 4:**
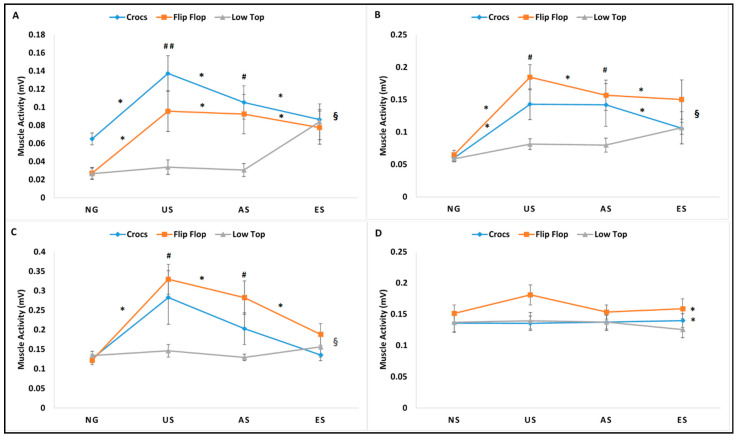
Mean muscle activity (mV) for vastus medialis (**A**), medial hamstrings (**B**), tibialis anterior (**C**), and medial gastrocnemius (**D**) during stance phase for Crocs, flip-flops, and low top slip resistant shoe during normal gait (NG), unexpected slip (US), alert slip (AS), and expected slip (ES) events. § denotes significant interactions between footwear type and gait/slip trial; * denotes significant difference for footwear across gait trials, and # denotes significant difference for gait trials across footwear. All differences were significant at the alpha level *p* < 0.05.

**Figure 5:**
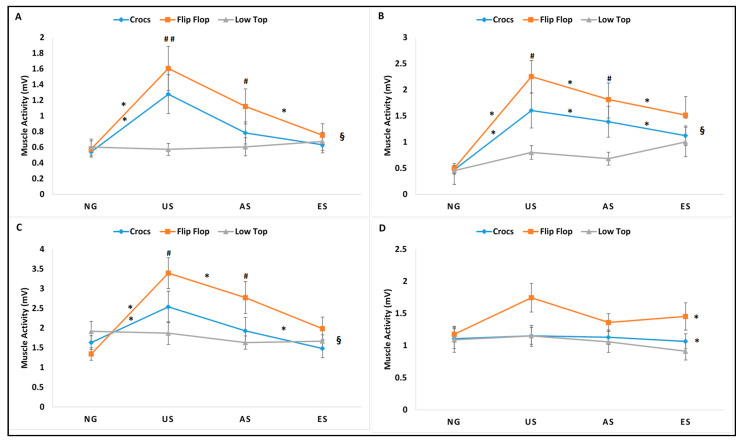
Peak muscle activity (mV) for vastus medialis (**A**), medial hamstrings (**B**), tibialis anterior (**C**), and medial gastrocnemius (**D**) during stance phase for Crocs, flip-flops, and low top slip resistant shoe during normal gait (NG), unexpected slip (US), alert slip (AS), and expected slip (ES) events. § denotes significant interaction between footwear type and gait/slip trial; * denotes significant difference for footwear across gait trials, and # denotes significant difference for gait trials across footwear. All differences were significant at the alpha level *p* < 0.05.

**Figure 6:**
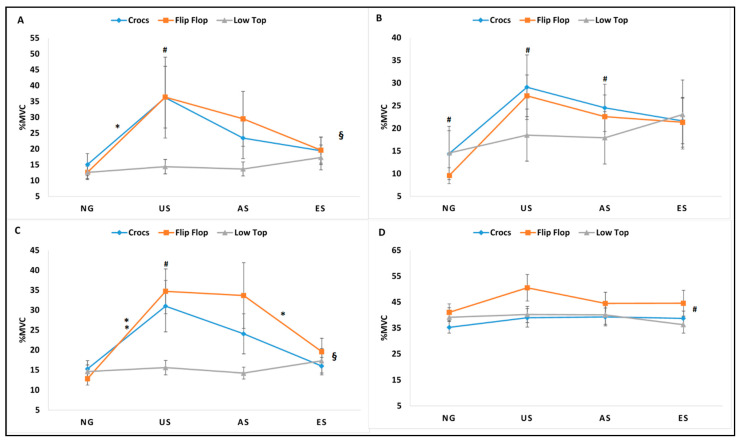
%MVC muscle activity for vastus medialis (**A**), medial hamstrings (**B**), tibialis anterior (**C**), and medial gastrocnemius (**D**) during stance phase for Crocs, flip-flops, and low top slip resistant shoe during normal gait (NG), unexpected slip (US), alert slip (AS), and expected slip (ES) events. § denotes significant interaction between footwear type and gait/slip trial; * denotes significant difference for footwear across gait trials and # denotes the significant difference for gait trials across footwear. All differences were significant at the alpha level *p* < 0.05.

The 3 × 4 within subjects repeated measures ANOVA revealed significant interactions between footwear and gait trials in mean muscle activity for VM, MH, and TA (mean VM, mean MH, and mean TA); in peak muscle activity for VM, MH, and TA (peak VM, peak MH, and peak TA); and in %MVC for VM and TA (%MVC VM and %MVC TA). A significant footwear main effect for mean muscle activity for MG (mean MG) and peak muscle activity for MG (peak MG) and significant gait trial main effect for %MVC for MH and MG (%MVC MG and %MVC MG) were identified. Results are reported as a repeated measures ANOVA table identifying the interactions and main effects for all dependent EMG variables with *p* value, F-statistic, and partial eta squatted effect sizes (Table 1). The significant interactions were followed with simple effects comparisons ignoring the main effects and for non-significant interactions, post-hoc pairwise comparisons of the significant main effect were performed. Descriptive statistics denoting significant simple effects for significant interactions and significant pairwise comparisons for the significant main effects are represented in Figure 4, Figure 5 and Figure 6 for all muscles (mean muscle activity: Figure 4A–D; peak muscle activity: Figure 5A–D; %MVC: Figure 6A–D).

## 4. Discussion

The impact of alternative footwear compared to slip-resistant footwear when exposed to unexpected, alert, and expected slips on the slip severity, kinematic, kinetics, and muscle activity during slip initiation phase has been previously reported. The purpose of this study was to analyze the impact of alternative footwear, (CC, FF) compared to LT on lower extremity muscle activity during non-slip and slip trials (NG, US, AS, ES) during the entire stance phase of the trials to gain insights into reactive and corrective muscle activation during such slip events. Significant interactions between footwear and gait trials existed for mean, peak, %MVC for VM and TA, and mean and peak for MH, suggesting the influence of both footwear and gait trial conditions in the outcome of lower extremity muscle activity. Results from the current analysis indicated significant differences in mean, peak muscle activity, and %MVC across all gait trials for the alternative footwear (CC and FF), while the LT had no significant differences across all gait trials either non-slip or slip trials, except for one variable (mean MH). On average, a greater magnitude of lower extremity muscle activity was seen in the slip trials, particularly the US and AS. The ES demonstrated similar muscle activity as the NG with no significant differences between them, suggesting that the individuals did not alter their muscle activity to maneuver an expected slippery flooring condition. The alternative footwear, particularly the FF, exhibited greater muscle activity compared to the LT during US and AS. The LT appeared to demonstrate the optimal performance in terms of low levels of muscle activity, both during non-slip and slippery gait trials with no significant differences across these gait trials. The main effect significance in footwear for mean and peak MH and the main effect significance in gait trials for %MVC MH also existed, indicating a greater magnitude of muscle activation during US and AS, in an attempt to recover from an induced slip, while LT and CC had significantly lower muscle activation compared to FF, which may be attributed to the footwear design features rather than the slip recovery, as they denote the main effect significance for footwear. Furthermore, it has been previously reported that footwear with elevated boot shafts that support the ankle may improve static balance performance [12] and that it may restrict joint range of motion and may hinder muscle activity around the ankle joint plantar flexors and dorsi-flexors. The MVCs from the four lower extremity muscles demonstrated no significant differences, supporting previous literature, as the footwear tested in this study did not have elevated boot shafts and did not hinder muscle activity. The observed significant differences in lower extremity muscle activity in the current footwear types during the stance phase of non-slip and slippery gait can be explained by both extrinsic (environmental) factors that include footwear design features and intrinsic (human) factors that include knowledge and anticipation of a slippery environment.

### 4.1. Extrinsic Factors–Impact of Footwear Design Characteristics

The geometric design of footwear has been shown to affect human balance and gait [12,14,15,16,17]. Few studies have focused on their impact on the use of alternative footwear such as the thong-styled flip flops and open-toed sandals on the biomechanics of human gait [18,19,21,28,29] and how the footwear affects the severity of slip related events [4,22,23,30]. Footwear serves as the interface between the human body and the supporting surface and can affect human balance and gait adversely [14]. Efficient transformation of the mechanical power output produced by the musculoskeletal system through the footwear is responsible for a good performance in gait. Hence, the design and type of the footwear becomes important in gait and posture. Based on the results from the current study, the footwear worn did not seem to affect any of the lower extremity muscles during baseline dry normal surface gait and expected slip conditions. No significant differences were seen during NG and ES across all footwear. However, during US and AS conditions, footwear differences affected the amount of muscle activity required to recover from slips. FF appeared to have the greatest amount of muscle activity, followed by CC, and finally the LT, requiring the least amount of muscle activity, emphasizing its better performance with the lowest incidence of slips and being efficient in requiring minimal muscle activity. The LT exhibited the least mean, peak, and %MVC for VM, MH, TA, and MG compared to both alternative footwear types (CC and FF) and across all gait trials. Although modifications in gait kinematics have been reported with the use of alternative footwear [18,28,29], the current study did not reveal differences in lower extremity stance phase during normal dry gait. However, the alternative footwear exhibited greater muscle activity and were least efficient during US and AS, while the LT proved to be the choice of footwear while maneuvering slippery flooring conditions.

### 4.2. Intrinsic Factors–Impact of Perception and Anticipation of Slips

Muscle activation during unexpected and anticipated slips have been studied previously [5,6]. The results from the current study support previous findings from Chambers and Cham [5], who reported a longer duration and great power muscle activity during hazardous slips compared to non-hazardous slips. Mean and peak muscle activity from VM, MH, and TA exhibited similar patterns of activation, with greater magnitude muscle activity in stance phase during US and AS, which represent the gait trials with a greater incidence of slips compared to NG and ES. Reactive strategies such as corrective responses by muscular forces are used to re-gain dynamic balance and are seen during unanticipated slips [4]. However, during an anticipated slip, proactive strategies are employed, which are best described as the balance control mechanisms that occur prior to an impending slip [5]. Muscular response strategies from the knee and hip have been normally related to the recovery from a slip with a smaller response coming from the ankle [4,5,31]. To recover from slips during US, higher magnitude muscle activity of the knee/upper leg muscles (VM and MH) could be required. The increased activity in VM may be attributed to the need to move the body center of mass over the base of support and accelerate the limb loading rate while the increased activity in MH may be attributed to the knee flexion moment commonly reported with anticipation during AS [5]. In addition, the greater activation of both the knee flexors and extensors may suggest a co-contraction between the agonist–antagonist pair of muscles. In contrast, the lower leg muscles (TA and MG) did not exhibit similar patterns as the upper leg musculature. The TA exhibited greater muscle activity in the stance phase of slippery gait trials, while there were no differences in MG muscle activity across trials. The increased activity in TA during the early stance phase have been related to a delayed achievement of foot-flat, which has been reported as an important aspect in slip recovery and gait continuation [25], while a null ankle moment during severe slips was also reported [26]. In the current study, the increased activity in TA was seen only in US and AS, which may be due to the reverse origin action of the TA to limit the forward movement of the foot and the leg after the initiation of the slip. The MG did not show an increased muscle activity in stance phase for slip trials, which may be attributed to the decreased stance phase push-off needed during a slip event. When participants were alert to the possibility of walking over a slippery surface (AS), an increased mean, peak, and %MVC for VM, MH, and TA were observed, supporting previous literature [5]. The anticipation of the slippery flooring condition during the ES condition exhibited similar muscle activity levels as the NG condition. The incidence of slips in the ES was significantly lower and suggests no extra requirement of muscle activity from the NG.

Based on the observed results from the current analyses, alternative footwear are not the optimal choice of footwear that an individual should wear, especially when exposed to a potentially slippery environment. The Crocs and flip-flops required a greater muscle activity response during the stance phase, especially when there was no anticipation of an impending slip in comparison to the slip-resistant shoes. Hence, alternative footwear such as Crocs, even though are very commonly used by nurses and doctors, should be avoided in slip-prone environments such as hospitals. Furthermore, these results can help in the design of alternative footwear, especially focusing on the sole tread pattern to minimize slip induced accidents.

Limitations to the study included the analysis of healthy young adults in an acute non-fatiguing situation. The current analyses are representative of healthy young adults and more research is warranted on different populations such as the elderly and clinical. Overexertion injuries have very high incidences for slip induced falls and makes the effort of recovering from an induced slip very demanding [32]. Hence, future research should focus on behavior of such alternative footwear and its impact on slips and slip induced falls when exposed to fatiguing workloads. Future research should assess muscle activity responses during non-fatigue conditions compared with fatigued conditions. Other limitations include the analysis of only the slipping leg (leg contacting the slippery contaminant) due the availability of the testing EMG system. Future research should also focus on EMG analysis of the trailing leg, as it plays a critical role in slip outcome and recovery.

## 5. Conclusions

In conclusion, greater lower extremity muscle activation during stance phase was seen in unexpected and alert slip conditions compared to normal dry gait and expected slip. In addition, footwear differences were seen for the alternative footwear (CC and FF) during US and AS, while the low top slip resistant shoe had no differences across all gait trials, suggesting that it is the most efficient footwear of choice, especially when maneuvering slippery flooring conditions, either with or without the knowledge of an impending slip. Although the alternative footwear is considered more comfortable and is easier to easier wear, they may not be the appropriate choice to improve muscular efficiency and prevent slips and slip induced falls.

## Figures and Tables

**Table 1:** Repeated measures analysis of variance (ANOVA) table for footwear and gait main effect and footwear × gait interaction.

Variable	Footwear Effect			Gait Trials Effect			Interaction (Footwear × Gait Trial)		
	*p* Value	F Statistic	*η*p2	*p* Value	F Statistic	*η*p2	*p* Value	F Statistic	*η*p2
Mean VM (mV)	0.002 *	F (2, 34) = 7.197	0.297	0.0005 *	F (3, 51) = 12.940	0.432	0.001 *	F (3.598, 61.171) = 5.662	0.25
Mean MH (mV)	0.008 *	F (2, 34) = 5.561	0.246	0.0005 *	F (3, 51) = 14.015	0.452	0.035 *	F (4.381, 74.476) = 2.661	0.135
Mean TA (mV)	0.0005 *	F (2, 34) = 9.656	0.362	0.0005 *	F (2.020, 34.345) = 10.503	0.382	0.022 *	F (2.451, 41.667) = 3.876	0.186
Mean MG (mV)	0.013 *	F (2, 34) = 4.972	0.226	0.306	F (3, 51) = 1.236	0.068	0.13	F (3.506, 59.604) = 1.901	0.101
Peak VM (mV)	0.002 *	F (2, 34) = 7.578	0.308	0.0005 *	F (2.080, 35.358) = 10.727	0.387	0.0005 *	F (2.869, 48.766) = 4.686	0.216
Peak MH (mV)	0.002 *	F (2, 34) = 7.239	0.299	0.0005 *	F (3, 51) = 16.650	0.495	0.032 *	F (3.362, 57.157) = 3.020	0.151
Peak TA (mV)	0.016 *	F (2, 34) = 4.655	0.215	0.0005 *	F (3, 51) = 12.091	0.416	0.001 *	F (3.400, 57.801) = 5.661	0.25
Peak MG (mV)	0.003 *	F (2, 34) = 6.847	0.287	0.086	F (3, 51) = 2.320	0.12	0.098	F (3.175, 53.977) = 2.181	0.114
%MVC VM	0.102	F (2, 34) = 2.441	0.126	0.008 *	F (1.925, 32.721) = 5.726	0.252	0.045 *	F (2.346, 39.890) = 2.979	0.149
%MVC MH	0.613	F (1.101, 18.709) = 0.298	0.017	0.0005*	F (2.061, 35.031) = 9.433	0.357	0.132	F (2.036, 34.617) = 2.145	0.112
%MVC TA	0.015 *	F (1.467, 24.933) = 5.773	0.253	0.0005 *	F (3, 51) = 10.181	0.375	0.007 *	F (2.739, 46.565) = 4.710	0.217
%MVC MG	0.077	F (2, 34) = 2.765	0.14	0.023 *	F (2.065, 35.099) = 4.140	0.196	0.135	F (3.168, 53.852) = 1.913	0.101

Note: VM—vastus medialis, MH—medial hamstring, TA—tibialis anterior, MG—medial gastrocnemius, MVC—maximal voluntary isometric contraction. * denotes significant difference at the *p* < 0.05 level.
